# Fabrication and Verification of Conjugated AuNP-Antibody Nanoprobe for Sensitivity Improvement in Electrochemical Biosensors

**DOI:** 10.1038/s41598-017-12677-w

**Published:** 2017-11-22

**Authors:** Patricia Khashayar, Ghassem Amoabediny, Bagher Larijani, Morteza Hosseini, Jan Vanfleteren

**Affiliations:** 1 Nanobiotechnalogy Department, Faculty of New Sciences & Technologies, University of Tehran, Tehran, Iran; 2 Center for Microsystems Technology, imec and Ghent University, Gent-Zwijnaarde, Belgium; 3 Osteoporosis Research Center, Endocrinology and Metabolism Clinical Sciences Institute, Tehran University of Medical Sciences, Tehran, Iran; 4 Department of Biotechnology, Faculty of Chemical Engineering, School of Engineering, University of Tehran, Tehran, Iran; 5 Nanobiotechnology Department, Research Center for New Technology in Life Sciences Engineering, University of Tehran, Tehran, Iran; 6 Endocrinology and Metabolism Research Center, Endocrinology and Metabolism Clinical Sciences Institute, Tehran University of Medical Sciences, Tehran, Iran

## Abstract

This study was designed to obtain covalently coupled conjugates as means for achieving higher stability and better coverage of the AuNPs by antibodies on the particle surface suitable for sensor performance enhancement. Starting by using a modified protocol, colloid gold solution, with mean AuNP core size of ~6 nm was synthesized. The protocol used for conjugation of AuNPs to osteocalcin antibody in this study relies on covalent and electrostatic attractions between constituents. Varieties of conjugates with varying combinations of crosslinkers and different concentrations were successfully synthesized. The obtained products were characterized and their properties were studied to determine the best candidate in sense of antibody - antigen reactivity. Using AuNP-GSH-NHS-Ab combination (1:1:1), the tertiary structure of the protein was maintained and thus the antibody remained functional in the future steps. This one-pot method provided a simple method for covalently coupling antibodies on the particle surface while keeping their functionality intact. The AuNP content of the solution also accelerated electron transfer rate and thus amplifies the detection signal. With the developed and discussed technique herein, a simple solution is modeled to be used for measuring serum levels of biomarkers in single and/or multiplexed sensor systems.

## Introduction

Designing sensors with higher efficiency depends on the development of novel materials to improve both the recognition and transduction steps. Studies have shown that labeling biomolecules with noble metal NPs results in highly sensitive and specific bioassays, while maintaining the bioactivity of the biomarker^1–3^. Such NPs can also help improve signal detection for low concentration of analytes.

Gold nanoparticles (AuNPs) are the most commonly used labels in sensor studies because of their intriguing size dependent electrical, optical, magnetic, and chemical properties as well as their ease of synthesis and surface functionalization^4,5^. Being redox active, AuNPs provide the possibility for miniaturizing the sensing devices to nanoscales, offering excellent prospects for chemical and biological sensing. They also provide a suitable platform for multifunctionalization with a wide range of organic or biological ligands for selective detection of biomarkers^6^.

As a result, broad potential of AuNPs for signal amplification in antibody (Ab) – antigen (Ag) reaction events on simple, ultrasensitive, multiplexed immunosensors become apparent^7^. AuNP conjugation with different proteins is well studied in the literature but the mechanisms behind the process are poorly understood^8–11^. Therefore, special attention is needed to ensure the quality of the end product. Different protocols were thus tested in this study to confer a controlled immobilization of antibody on the surface, while maintaining the activity of the bounded antibody. With the developed and discussed technique herein, a simple solution has been modeled to be used for measuring serum levels of biomarkers in single and/or multiplexed sensor systems^12,13^.

## Results and Discussion

### Characterization of AuNPs

The sodium citrate in the AuNP preparation procedure behaves as a reducing agent. It also provides the negative surface charge around the AuNPs, needed to repel the particles and restrain any aggregation. The concentration of gold salt and trisodium citrate as well as the temperature and mixing rate are main factors defining the size-distribution of the synthesized colloidal gold nanoparticles^14^.

**Figure 1:**
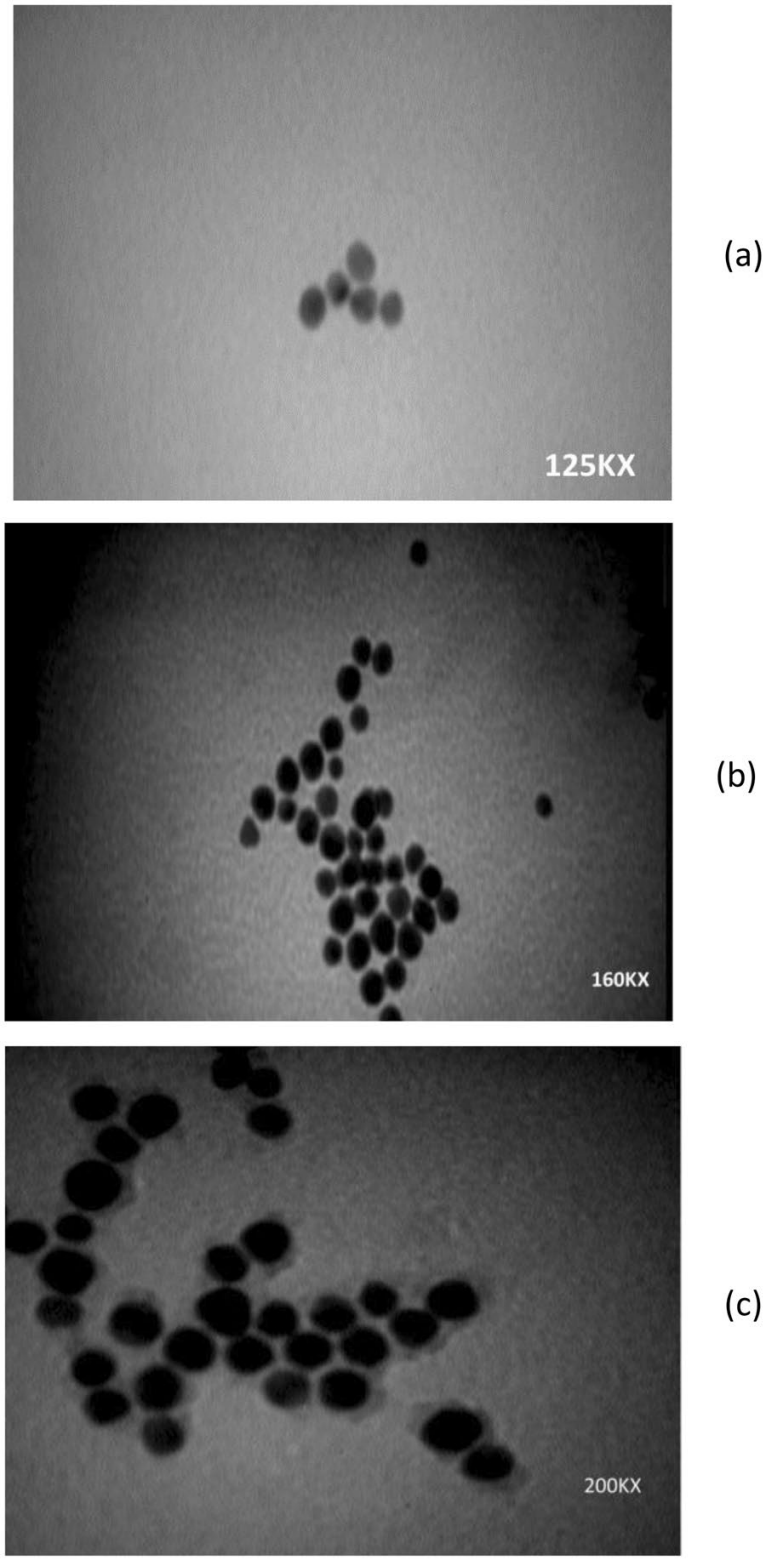
TEM results. (**a**) Monodispersed gold nanoparticles, (**b**) Gold nanoparticles capped with GSH, (**c**) Antibody- conjugated gold nanoparticles (With permission)^13^.

In this work, TEM was used to characterize the morphology and determine the size of the as-prepared AuNP solution. According to the ImageJ results, uniformly distributed, mainly spherical, nanoparticles with an average size of 6.53 ± 1.8 nm were observed in the solution with very few particles of higher and lower size distribution (Fig. 1).

**Figure 2:**
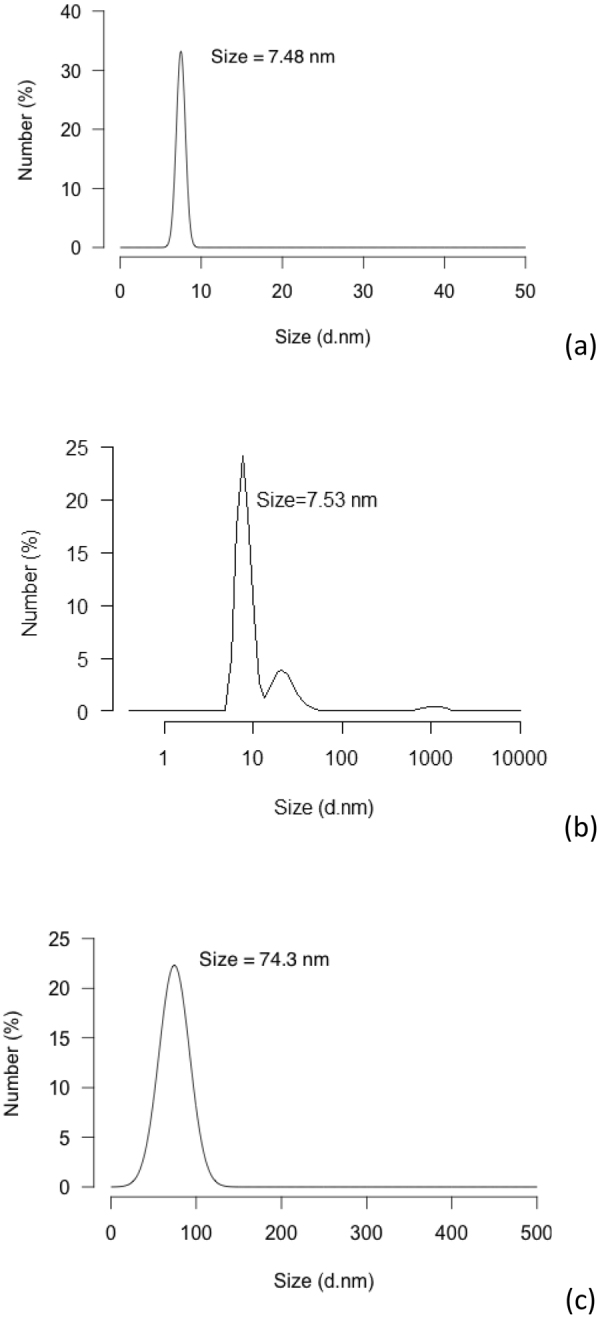
DLS results. (**a**) Gold nanoparticles, (**b**) Gold nanoparticles capped with GSH, (**c**) Ab - conjugated gold nanoparticles (with Permission)^13^.

The mean hydrodynamic diameter of the nanoparticles, suggestive of the mean diameter of the majority of the particles according to number (%) was measured using a zeta-sizer nano-ZS (Fig. 2). As expected, the DLS values were larger than those reported by TEM (7.48 nm vs. 6.53 nm) due to the presence of the double layer effect in the calculations of the hydrodynamic radius of the particles in the solutions, and its absence in that of the dried-state samples imaged by TEM. Moreover, DLS results are quite dependent on the viscosity and temperature.

Assuming a spherical shape and a uniform FCC crystal structure, the average number of gold atoms per nanoparticle (N) was calculated using the following equation (eq. 1) and based on TEM findings:

1 $$N=\frac{\pi }{6}\frac{\rho {D}^{3}}{M}=8594$$

where D is the average core diameter of the particles (nm), ρ is the density for fcc gold (19.3 g/cm^3^), and M stands for the atomic weight of gold (197 g/mol)^15^.

Then the molar concentration of the nanosphere solution was calculated by dividing the total number of gold atoms (N_total_ = initial amount of gold salt added to the reaction solution) by the average number of gold atoms per nanosphere (N), using the following equation (eq. 2):

2 $$C=\frac{{N}_{Total}}{NV{N}_{A}}=1.96\ast {10}^{-6}mol/L$$

**Figure 3:**
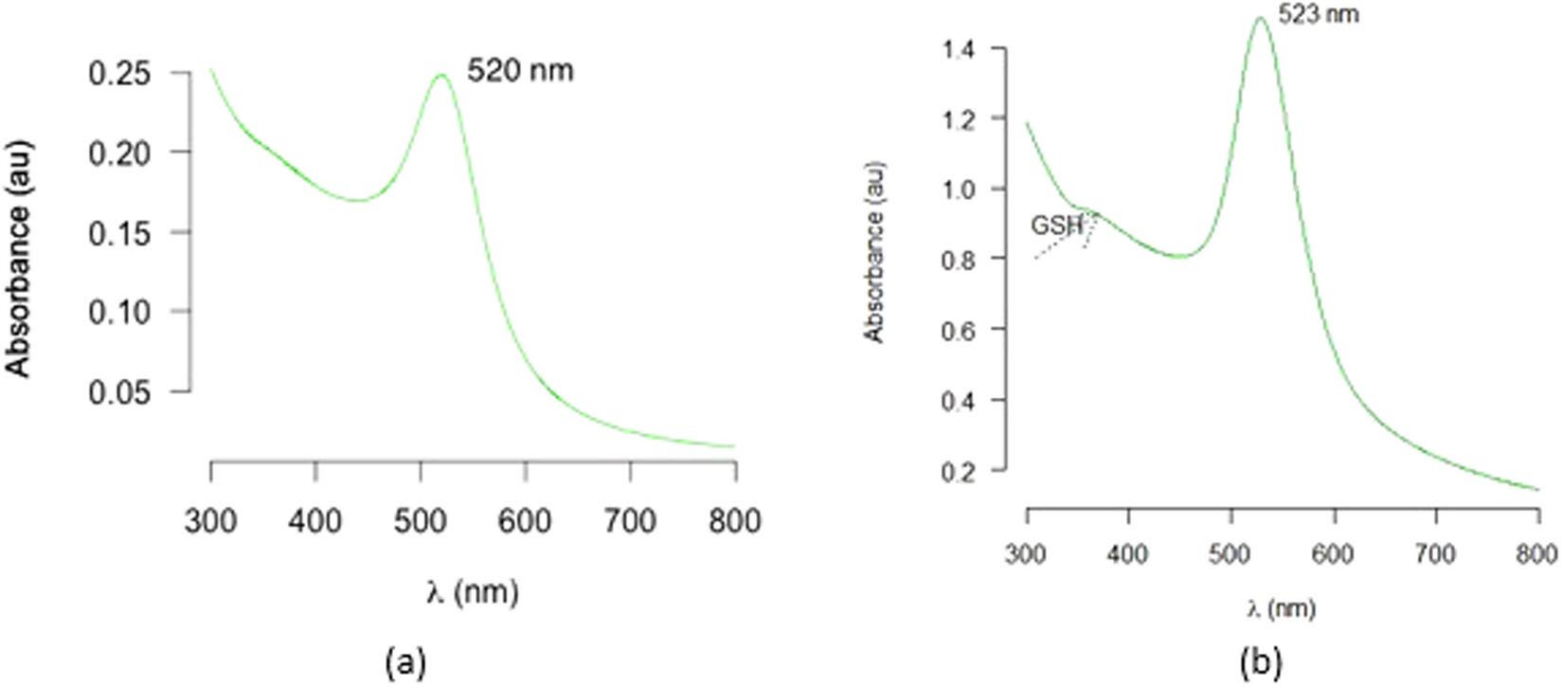
UV–Vis spectra of (**a**) colloidal AuNPs (**b**) GSH-capped AuNPs.

The properties of the prepared AuNP solution were also determined through calculation of its extinction coefficient as well as the absorption peak and curve appearance. The UV–Vis spectrophotometer has been the first optical technique employed for this regard. In this method, the width of the absorption spectra is related to the size distribution range of the nanoparticles; as a result, the absorption peak shifts to longer wavelengths with increasing particle size. In the present study, the plasmon absorption peak was observed at ∼520 nm, which is corresponding to the typical surface plasmon resonance (SPR) of colloidal AuNPs prepared by the conventional citrate reduction method (Fig. 3)^16^. Lack of a band at around 680 nm indicated the absence of aggregated gold particles and thus confirmed the successful preparation of an AuNP solution^17^.

The Lambert-Beer law was used to calculate the extinction coefficient, the sum of absorption and scattering coefficients (ε), of the nanoparticle solution (eq. 3).

3 $$A=\varepsilon bC\to \varepsilon =2.65\ast {10}^{8}M/cm$$

Where A is the absorbance at 520 nm, b is the length of solution that the light passes through (cm) and C is the concentration of the solution (mol/cm^3^).

It should be noted that the calculated extinction coefficient of our colloid solution was higher than the solutions made using the same technique in the literature (8.56 ∗ 10^6^ or 5.14 ∗ 10^7^)^15^.

### AuNP/Ab nanocomplex

This study was designed to obtain covalently coupled conjugates as means for achieving higher stability and better coverage of the AuNPs by antibodies on the particle surface suitable for sensor performance enhancement. GSH is a tripeptide biomolecule (–Glu-Cys-Gly), containing a –SH group which can easily be adsorbed onto the AuNP surface. A dense layer of GSH-AuNP enlarges the electroactive surface area and provides carboxylic groups for covalently binding of the particles to a dense layer of antibody/protein for detection. EDC and sulfo-NHS, on the other hand, acted as cross-linkers facilitating the bonding of an amine group of the antibody to the carboxylic group of GSH.

**Figure 4:**
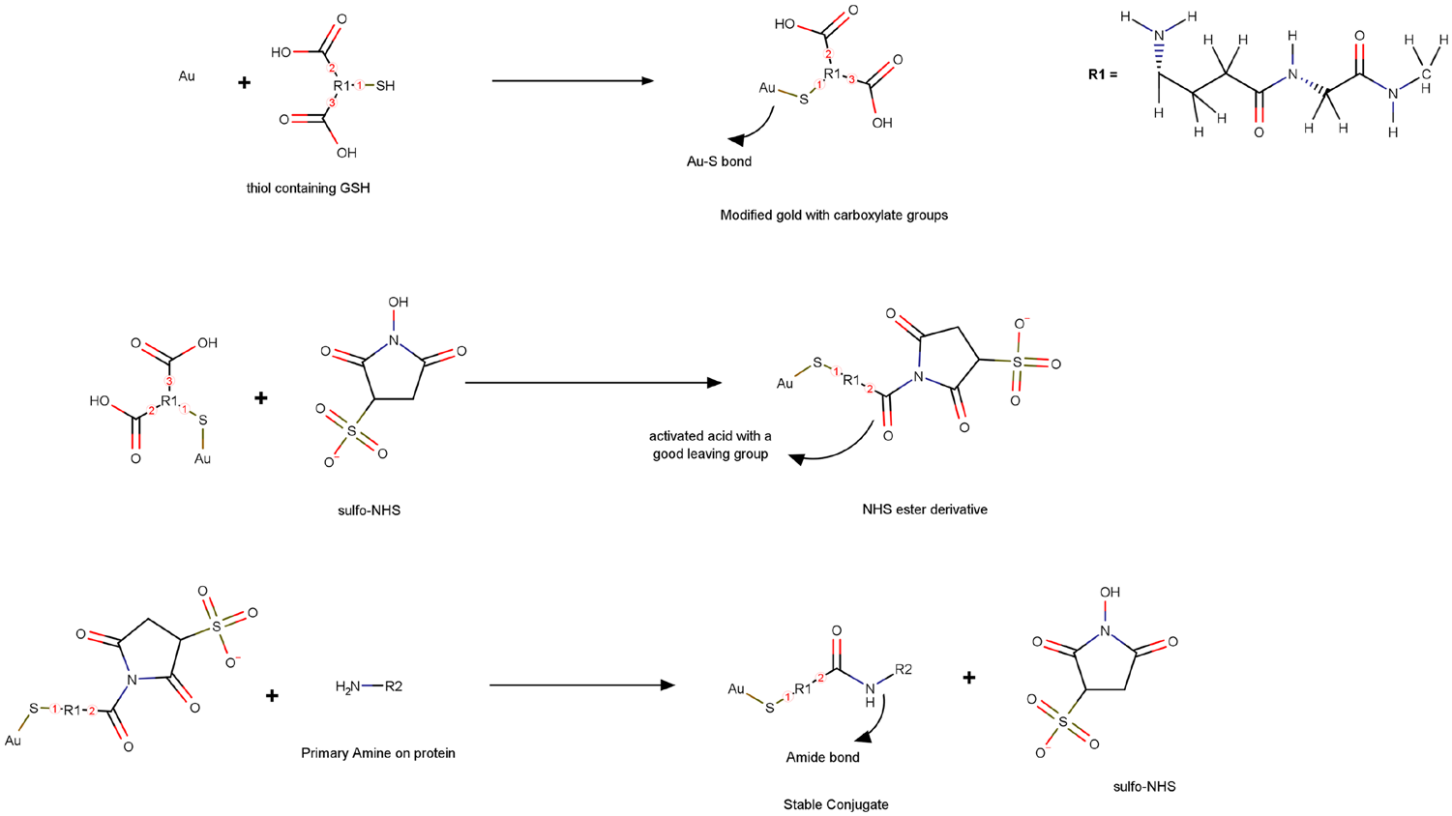
Representative schematic showing nanoconjugate formation steps.

In other words, using this combination, the binding of antibody complex to gold atoms happened through thiol linkages as well as ionic interactions between the negatively charged gold surface and the positively charged terminals on the complex, along with interactions originating from absorption of hydrophobic groups on AuNPs (Fig. 4). This is while the conjugates developed through direct absorption of the antibody on the surface of the nano-colloid particles using non-covalent bonds, such as London–van der Waals force and hydrophobic interactions, are reported not to be stable^18,19^.

### Functionality of AuNP/Ab nanocomplex

As mentioned earlier, the final goal in the sensor development process is to maintain the bioactivity and functionality of the antibody immobilized on the electrode surface. In this regard, various techniques are reported in the literature^20–22^. Our results revealed that using AuNP-GSH-NHS-Ab combination (1:1:1), the tertiary structure of the protein was maintained and thus the antibody remained functional in the future steps. This was confirmed based on a significant decrease noted in the maximum peak after the antigen was left to react with the electrode modified with the 1:1:1 GSH-NHS-Ab nanoconjugate for at least 2 hours. The reason behind the noted decrease in the measured current is the fact that the binding of antigen to the immobilized antibody disrupts electron transfer.

**Figure 5:**
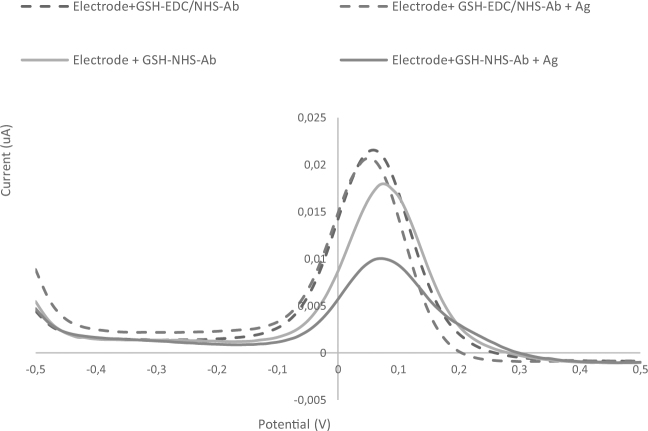
DPV plots of two different nanoconjugates before and after reaction with antigen (Ag) for 2 hrs.

As for the rest, a minimum change from baseline value (<3%) was reported, indicating that the antibody bounded using those protocols either had lost its activity or did not have the correct orientation (Fig. 5). In other words, the conjugates were found not to be functional in the presence of EDC; this could be explained by the fact that EDC may resemble hydroxyapatite effects and thus alter the tertiary structure of the osteocalcin^23^.

Another advantage of the abovementioned method was that covalent coupling via a cross-linker is specific and controllable. Thus, the nanocomplex prepared using this protocol is going to be discussed in the rest of this article.

### Characterization of AuNP/Ab nanocomplex

Characterization of the resulting conjugates was done by TEM measurements of freshly prepared solution (Fig. 1). GSH molecules are believed to form certain intermolecular hydrogen binding among themselves, even when they are adsorbed on AuNPs^24^. The concentration ratio of GSH and AuNP, thus, is one of the important factors that affects the aggregation rate in the solution. According to our results, no aggregates were formed upon the addition of GSH to the AuNP solution. UV-Vis confirmed the successful formation of GSH-capped AuNPs; and the absence of any significant alteration in particle morphology or size, based on TEM and DLS findings, confirmed the latter (Figs 2 and 3).

The attachment of the antibody to GSH-modified gold nanoparticles, on the other hand, resulted in a considerable increase in size and the formation of core-shell structure, which is in accordance with the findings of previous reports^25,26^. In other words, the size increase confirmed the existence of an additional layer of conjugated antibody on the nanoparticles.

Assuming that the increase in the particle size (diameter) was caused by the NHS-Ab, one could also conclude that this increase is equivalent to the diameter of the monolayer of antibody around the core AuNP. Thus, the volume of the core-shell is calculated as the difference between the antibody-conjugated nanoparticles and the AuNP, given by (eq. 4):

4 $$V=0.5236({D}^{3}-{d}^{3})=8333.53\,n{m}^{3}$$

where D is the diameter of the antibody-conjugated nanoparticles and d is the diameter of the core AuNP. By dividing the volume of the core-shell by that of the antibody obtained using the calculated hydrodynamic diameters, it could be concluded that at least 2 antibodies attach to each nanoparticle in this technique.

Nanoparticle colloid is stabilized by the electrostatic forces, indicated by the zeta potential of the particles in solution. This arises due to an energy barrier originating from the balance between two opposing forces: the van der Waals attractive (V_A_) and electrical double layer repulsive (V_R_) forces between the particles approaching each other^27^. This barrier prevents the two particles from agglomerating.

Zeta potential is influenced by the thickness of the electrical double layer, the electrical charge in this layer and dielectrical constant^28^. As a result, the particle’s surface charge is an important factor influencing the stability and reactivity of the nanoparticle, and thus can greatly enhance the nanoparticle conjugates’ application. In line with the Huckel’s theory, our results showed that the zeta potential value increased with particle size, and that the particle charge is directly proportional to the zeta potential.

### Optimization of AuNP/Ab nanoconjugate

The preparation of a well-dispersed nanoprobe with uniform size distribution and no aggregation is of great importance in immunoassay studies. Therefore, lowest amount of antibody enough to stabilize the gold was determined by incubating 1 mL of colloid gold with antibody solutions at varying concentrations (1–50 ug/mL, 1 mL) for 5 min. Thereafter, 0.5 mL of 10 % (w/v) NaCl was added and the color of the solution was noted. The concentration in which the color of the solution turned blue was considered as the minimum required amount for stabilization.

Further tests to assess the reactivity of the nanocomplex towards antigen showed that lower concentrations of antibody produced small current change, suggestive of the absence of sufficient amount of antibody to react with the antigen on the surface. Higher concentrations of the nanoprobe, on the other hand, improved the current while contributing to higher nonspecific adsorption.

As mentioned in the literature, environmental factors such as temperature and pH play an important role in the efficiency of the nanoconjugate^29,30^. Thermal optimum depends on the chemical nature of the protein, adding that hydrogen bonds are more stable at low temperature, whereas the strength of the hydrophobic bonds increases with temperature^31^. Antigen - antibody reactions are also expected to stabilize at lower temperatures. According to our results, however, no significant change was reported when the incubation was performed at a temperature range of 4 °C to room temperature. As a result, to simplify the process, all the processes were performed at room temperature and the optimized incubation time for this temperature, needed for saturated binding of the immunoreaction, was calculated to be 2.5 hours.

The pH of the solution is another factor affecting the quality of the end product. The optimal reaction rate of NHS chemistry proceeds around pH 4.5–7.2. In other words, NHS-esters hydrolyze faster in higher pH, whereas no reaction takes place at pH values lower than 3.5^32^. As for GSH, best results are achieved by adjusting the pH at values in which the carboxylic groups are in their protonated form. This occurs when GSH is as a zwitterion (pH = 3–7)^33^. Moreover, extreme pH values induce marked conformational changes in the Ab molecule that probably may affect its complementarity with the antigen. On the other hand, since osteocalcin, the Ab used in this study, has an isoelectric point (pI) of 4.4, at lower pH values it carries more positive charge and thus positively influences the electrostatic attraction between the oppositely charged surface carboxyl anions and the protonated amino group of the ligand. As a result, no attempts were made to change the negative pH of the sulfo-NHS solution.

## Conclusion

Generally, electrochemical sensors have the virtue of high sensitivity and selectivity, but they require several fabrication steps. Through the inherent interaction between AuNPs and antibody biomolecules, a novel gold nanoprobe was developed herein to be used in nonenzymatic electrochemical immunoassays. This one-pot method provides a simple method for covalently coupling antibodies on the particle surface while keeping their functionality intact. The AuNP content of the solution also accelerates electron transfer rate and thus amplifies the detection signal.

The nanoprobe properties depend on particle size, combination and concentration of cross-linker and the Ab on one hand and the immobilization technique on the electrode on the other hand. The main idea of this study was to optimize the nanoconjugate to have an immunologically reactive solution toward the antigen. The next step, thus, would be to optimize the immobilization process of the nanoprobe on the electrode surface. Considering the results, the proposed method shows a promising potential for clinical applications.

## Methods

### Materials and reagents

Osteocalcin monoclonal antibody (ab13418) and full-length osteocalcin protein (ab152231) were purchased from Abcam Co. (UK) and were reconstituted in phosphate buffered saline (PBS). The latter was purchased in powder form from Sigma-Aldrich Chemical Co. (Belgium) and was used to prepare a working solution of 10 mM phosphate buffer (NaCl 0.138M, KCl 0.0027M), pH 7.4 at 25 °C. Tetrachloroauric acid (HAuCl_4_·3H_2_O), sulfo-a-N-hydroxysuccinimide (sulfo-NHS), b-1-ethyl-3-(3-dimethylamonipropyl) carbodiimide (EDC), L-Glutathione reduced (GSH), Bovine Serum Albumins (BSA), Potassium hexacyanoferrate (III) (K_3_Fe(CN)_6_) and trisodium citrate were also obtained from Sigma-Aldrich. Distilled water was used throughout the experiments.

Carbon (DRP-110) screenprinted electrodes (SPEs) (DRP-C223AT) were purchased from Dropsens (Spain).

### Apparatus

In order to obtain information about structure, morphology and size of the synthesized AuNPs and antibody conjugated AuNPs, UV-Vis spectroscopy, transmission electron microscopy (TEM) analysis, dynamic light scattering (DLS) and zeta potential measurements were applied.

The absorption spectroscopic measurements were recorded using a UV Visible Spectrophotometer SPECORD 250 (Analytik Jena, Japan).

The TEM images were obtained with an EM10C 80 KV (Zeiss, Germany) transmission electron microscope. The samples for TEM measurements were prepared by placing a droplet of the colloidal solution onto a carbon-coated copper grid and allowed it to dry in air. Using ImageJ software, the average core diameter of the particles were measured.

DLS measurements to determine the size of the particles were carried out using a MALVERN Instrument MAL1001767 UK, at the temperature of 25 °C and pH 7.4. By this method, the dynamics of the particles system in relation with the Brownian motion and fluctuations of scattered light was investigated with time. The frequency shifts, the angular distribution, the polarization, and the intensity of the scatter light were determined by the size, shape and molecular interactions in the scattering material. The Malvern Zetasizer Nano Instrument was also used for all zeta potential measurements.

All electrochemical experiments were performed using a computer-controlled DropSens STAT 400 (DropSens, Spain). They were carried out in a beaker, at room temperature (23 °C), using the three-electrode configuration. Differential Pulse Voltammetry (DPV) was performed to confirm surface modification changes. The DPV cycles were performed in 0.1 mM K_3_[Fe(CN)_6_], containing 0.01 M NaCl solution, applying following parameters: 0.05 V modulation amplitude, 0.01 s modulation time, 0.01 V step potential and voltage range from −0.5 to 0.5 V.

### Synthesis of conjugated AuNP - antibody nanoprobe

#### — Synthesis of AuNPs

In order to synthesize 10–100 nm AuNPs, the Turkevich method improved by Frens was used^34,35^. Briefly, 200 ml of 0.01 % HAuCl_4_ solution was brought to boil while stirring gently. Thereafter, 4.5 ml of 1 % trisodium citrate was rapidly added to the boiling solution while stirring constantly for an additional 15 min. Once mixing occurred, the color of the solution changed gradually from light yellow to wine red. The solution was then cooled down to room temperature. This solution was stored at 4 °C for further use.

#### Synthesis of GSH-capped AuNPs

A stock solution of glutathione (10 mM) was prepared. Dilute HCl solution (10 mM, 100 mL) was added to colloidal AuNP solution (3 mL) to lower the pH (~4)^36^. An aliquot of GSH solution (10 mM, 30 mL) was added to the acidic gold solution. This was the maximum amount required before the color of the solution changed from deep red to blue, indicating formation of aggregates amongst AuNPs.

#### Synthesis of AuNP - antibody Conjugates

A design of experiments (DOE) was prepared to assess the role of various parameters (different crosslinker combinations and concentration ratios) in manufacturing the AuNP - Ab conjugates, which would result in the best performance for the nanoprobe. The four parameters studied were the EDC (0, 100 mM, 200 mM, and 400 mM), sulfo-NHS (0, 100 mM, 200 mM and 400 mM), GSH (0, 10 mM) and Ab concentrations (10 µg/mL and 20 µg/mL). In other words, different concentration ratios of these material were combined to fabricate the nanoconjugate.

In each protocol, specific concentrations of the freshly prepared EDC and sulfo-NHS solutions were mixed and left to react for half an hour. Then the Osteocalcin antibody was added to the solution and incubated for 2.5 hours. The complex was finally added to the GSH-capped AuNP solution and left to react for another hour. After each step, the solution was mixed using a fixed-speed vortex mixer. All the above mentioned procedures, mixing and incubating, was done at room temperature.

### Functionality of AuNP/Ab nanocomplex

The reactivity of the AuNP/Ab nanoconjugates prepared using different protocols was tested using osteocalcin protein, herein referred to as the antigen (Ag). This was done by dropping 3 µL of the AuNP/Ab nanoconjugates on a gold SPE and waiting for an hour to dry. 20 µl of 0.2% BSA solution in PBS buffer was added to block the unreacted active functional groups. The electrode was then rinsed for 3 min with 0.1 M PBS to eliminate non-specific bindings. The baseline DPV plots were then recorded in 0.1 mM K_3_[Fe(CN)_6_] for each nanoconjugate.

Thereafter 3 µL of the Osteocalcin antigen was dropped on the modified electrodes and left to react for 0.5, 1, 2, 5, 10 and 20 hours. The second DPVs were then plotted.
